# Participatory Development and Assessment of Audio-Delivered Interventions and Written Material and Their Impact on the Perception, Knowledge, and Attitudes Toward Leprosy in Nigeria: Protocol for a Cluster Randomized Controlled Trial

**DOI:** 10.2196/53130

**Published:** 2024-01-24

**Authors:** Ngozi Murphy-Okpala, Tahir Dahiru, Anna T van ’t Noordende, Carolin Gunesch, Joseph Chukwu, Charles Nwafor, Suleiman Hudu Abdullahi, Chukwuma Anyaike, Ugochinyere Angelic Okereke, Anthony Meka, Chinwe Eze, Okechukwu Ezeakile, Ngozi Ekeke

**Affiliations:** 1 RedAid Nigeria Enugu Nigeria; 2 Leprosy and Tuberculosis Relief Initiative Jos Nigeria; 3 NLR International, The Netherlands Amsterdam Netherlands; 4 DAHW-German Leprosy and TB Relief Association Wurzburg Germany; 5 National Tuberculosis and Leprosy Control Programme, Federal Ministry of Health Abuja Nigeria; 6 Department of Art Education University of Nigeria Nsukka Nigeria

**Keywords:** audio health education, community perception of leprosy, health education, leprosy, Nigeria, persons affected by leprosy

## Abstract

**Background:**

In Nigeria, similar to many leprosy-endemic countries, leprosy is highly stigmatized. High levels of stigma among community members as well as internalized stigma among persons affected by leprosy often result in negative psychosocial consequences for those affected. To break this vicious cycle, it is important to conduct context-specific behavioral change activities. Although written material has been successful in improving knowledge and perception, it is not suitable for populations with low educational levels. Audio-delivered interventions are likely to be more suitable for people who are illiterate. This study proposes to assess the impact of an audio-delivered intervention on the perception (knowledge, attitudes, and beliefs) of community members with regard to leprosy in Nigeria.

**Objective:**

This study aims to assess the impact of audio-delivered and written health education on the perception of leprosy. Specific objectives are to (1) investigate the perception (local beliefs, knowledge, and attitudes) of community members toward leprosy and persons affected by leprosy; (2) investigate whether there is a difference in impact on perception between participants who have received audio-delivered health education and those who have received written health education, with specific reference to gender differences and differences between rural and urban areas; and (3) assess the impact of the participatory development of the audio-delivered and written interventions on empowerment and internalized stigma of persons affected by leprosy who developed the interventions. Additionally, we will translate and cross-culturally validate 4 study instruments measuring outcomes in 2 major Nigerian languages.

**Methods:**

We will use a mixed methods, cross-sectional study design for the intervention development and a 3-arm cluster randomized controlled trial for its implementation and evaluation, comprising (1) baseline assessments of knowledge, attitudes, perceptions, and fears of community members, to develop the audio-delivered content and written material, and the self-esteem and internalized stigma of persons affected by leprosy; and (2) participatory development of the audio-delivered content and written material by persons affected by leprosy and the pilot and implementation of the interventions. This will be done among different groups (selected using cluster randomization) that will be compared (control group, audio-intervention group, and written material group) to evaluate the intervention and the impact of developing the intervention on the persons affected.

**Results:**

This study was funded in June 2022, and community member participant recruitment started in January 2023. Baseline data collection was completed by May 2023 (n=811). Participatory cocreation of the audio and written health education content began in July 2023, and the materials are currently under development. Study results are expected in September 2024.

**Conclusions:**

Study findings will contribute to developing evidence-based, context-specific behavioral change interventions, which are critical to addressing stigma in many leprosy-endemic communities where leprosy is highly stigmatized, and contribute toward global triple zero leprosy efforts.

**Trial Registration:**

Pan African Clinical Trial Registry PACTR202205543939385; https://pactr.samrc.ac.za/TrialDisplay.aspx?TrialID=23667

**International Registered Report Identifier (IRRID):**

DERR1-10.2196/53130

## Introduction

### Overview

Leprosy is an infectious disease caused by *Mycobacterium leprae* [1]. Leprosy has been stigmatized since ancient times [2]. Stigma refers to a negative social response from one group toward a low-power, stigmatized group [3]. Stigma is “a social process, experienced or anticipated, characterized by exclusion, rejection, blame, or devaluation that results from experience, perception, or reasonable anticipation of an adverse social judgment about a person or group” [4]. Stigma can occur at different levels, for example, at the intrapersonal, interpersonal, community, institutional, and structural levels [5]. Many people affected by leprosy experience the negative consequences of their condition [6,7]. Stigma can have a very negative effect on employment and education opportunities, social interaction, housing, and access to health care [8]. Stigma can also indirectly cause stress and negatively impact mental well-being, quality of life, and physical health [8]. Stigma can delay seeking treatment [9] and can also impede adherence to treatment [10]. Family members and friends may experience courtesy stigma (stigma by association) [11,12].

Perception, which refers to how individuals or groups “see” an object, person, event, or institution [13-15], is an important driver of stigma [4]. The origin of stigma lies in public perceptions about people who are stigmatized. Perception comprises knowledge, beliefs, and attitudes, which are in turn influenced by personal factors (eg, personality and experience) and environmental factors (eg, culture and religion) [13,14]. Leprosy-related stigma is mainly caused by fears of contagion, external manifestations, and disabilities; religious and cultural beliefs; misconceptions; and a lack of knowledge [2,16]. Personal characteristics such as age, gender, occupation, education, and living area have also been associated with leprosy-related community stigma [17-20]. Stigma reduction (which often consists of positively influencing the perception of leprosy and increasing knowledge of leprosy) is crucial to improving the lives of people affected by leprosy and to improving leprosy services. Several interventions have successfully reduced leprosy-related stigma [21-24]. Interventions that are culture-specific and contextualized tend to be more effective [25,26].

Nigeria is among 23 global priority countries identified by the World Health Organization (WHO). In 2019, a total of 2424 new patients with leprosy were detected in Nigeria, 15% of whom had Grade 2 disabilities (G2Ds) [27]. Nigeria is among the few countries that reported more cases of G2Ds in 2019 than in previous years [27]. G2D among new patients is used as an indicator of late detection of leprosy. In addition, G2Ds (visible impairments) often exacerbate stigma and discrimination [28,29]. Leprosy is a stigmatized disease in Nigeria [30,31]. In a study among persons affected by leprosy and community members in Western Nigeria, the stigmatization of leprosy was mainly linked to perceived infectivity and perceived immoral behavior [31].

Despite progress in the control of leprosy in the last decades, the disease remains highly stigmatized [7], especially in Nigeria [30,31]. High levels of community stigma as well as internalized stigma among persons affected by leprosy may result in delayed diagnosis and disabilities [32]. To break this vicious cycle, it is important that context-specific attitude and behavior change activities are carried out. From other studies, we know that changing perceptions and improving knowledge can lead to behavior change [18,33,34]. For example, between March and June 2020, several printed materials that aimed to improve the perception of leprosy and increase knowledge about leprosy were evaluated in Uttar Pradesh, India. These context-specific materials were developed as part of NLR International’s Post-Exposure Prophylaxis project. Analysis revealed an association between the number of posters seen and a positive change in knowledge and stigma scores [35].

Access to appropriate health information is an essential step in the fight against stigma and discrimination. However, written materials are not suitable for populations with low educational levels [36]. To make health information accessible to people who are illiterate in many leprosy-endemic communities of the global south (especially women and girls), it is important to provide information through modalities other than printed materials. It is believed that audio-delivered interventions would be more suitable for people who are illiterate and low-literate. Audio-delivered interventions, for example, radio, have been shown to be successful for different stigmatized conditions such as HIV, leprosy, and albinism [37-39].

This study aims to assess the impact of an audio-delivered intervention on the perception (knowledge, beliefs, and attitudes) of community members regarding leprosy by comparing an audio-delivered intervention with written health education. The study will be conducted in Nigeria.

### Primary and Secondary Objectives

Our primary objective is to assess the impact of audio-delivered and written health education on the perception of leprosy in Nigeria. Additionally this study has the following secondary objectives: (1) to investigate the perception (local beliefs, knowledge, and attitudes) of community members toward leprosy and persons affected by leprosy; (2) to investigate whether there is a difference in impact on perception between participants who have received audio-delivered health education and those who have received written health education, with specific reference to gender differences and differences between rural and urban areas; and (3) to assess the impact of the participatory development of the audio and written interventions on empowerment and internalized stigma of persons affected by leprosy who developed the interventions.

## Methods

### Study Design

We will conduct a cross-sectional study (intervention development) and a 3-arm cluster randomized controlled trial (RCT; intervention implementation and evaluation). The three arms consist of (1) an intervention group who will receive the audio-delivered intervention, (2) an intervention group who will receive the written intervention (poster or flyer), and (3) a control group who will not receive any intervention.

### Study Location and Setting

The study will be carried out in 6 local government areas (LGAs), 3 each in Cross River State (Boki, Calabar-South, and Obubra) and Taraba State (Jalingo, Yorro, and Zing). The 3 LGAs per area will be selected based on similarity in terms of literacy rate and prevalence of leprosy. Cross River is located in southern Nigeria, while Taraba is in the north. The total population of the study area is 940,540, estimated from the 2006 census, with 542,494 in Cross River and 398,046 in Taraba states. Both states have been selected because of the high prevalence of leprosy and G2D.

Cross River State notified a total of 106 new cases (17% with G2D) in 2019. The most common language spoken in Cross River State is Nigerian Pidgin. Over 90% of the people living in the selected LGAs in southern Nigeria speak Nigerian Pidgin. Other languages spoken in Cross River State include Efik, Ekoi, and Yala. The illiteracy rate (those with no schooling or primary or secondary education who cannot read at all) is 12.2% and 26.4% for male and female individuals, respectively. Occupations include farming, trading, and civil service employment. About 58% of women own mobile phones, compared with 71% among men [40]. Cross River State’s poverty index is 0.146, and the poverty headcount rate is 36.3%. The case notification rate for leprosy is 2.76 per 100,000 people based on 2020 data.

Taraba is among the 15 high-burden states for leprosy in Nigeria; an average of 100 new cases were detected between 2013 and 2017, with an average of 6% and 5% of children and G2D cases, respectively. This figure is likely to be underreported, considering the significant presence of isolated populations such as nomadic pastoralists and internally displaced people in the state.

The most common language spoken in the selected LGAs in Taraba State is Hausa; 80% to 90% of the people living in the selected LGAs in Northern Nigeria speak Hausa. Other languages spoken in Taraba State include Mummuye and Fulfulde. The illiteracy rate is 30.1% and 64.9% in Taraba State for male and female individuals, respectively. Occupations in Taraba include farming, mainly crop production and cattle rearing, petty trading, and civil service employment. The majority of men (71%) own a mobile phone, while only 44.6% of women do. Taraba State’s poverty index is 0.448, and the poverty headcount rate is 87.7%. The case notification rate for leprosy is 2.30 per 100,000 people.

### Study Population

The following 2 groups of participants will be included in the study: persons affected by leprosy (for the participatory development of the interventions) and community members (the target group of the interventions).

### Inclusion and Exclusion Criteria

Individuals aged 18 years or older will be included in this study. Individuals who do not speak Nigerian Pidgin or Hausa and who are unable or unwilling to give informed consent will be excluded from the study.

### Study Duration and Sample Size Calculation

This study’s duration will be 2 years. The sample size for the various components of the study is as follows. (1) A total of 200 community members (100 for each language per study area) will be included in the cross-cultural validation of the Explanatory Model Interview Catalogue Community Stigma Scale (EMIC-CSS) and Social Distance Scale (SDS), while 100 persons affected by leprosy (50 for each language per study area) will be included in the cross-cultural validation of the Internalized Stigma of Mental Illness (ISMI) scale and Rosenberg Self-Esteem Scale (RSES; see below). (2) A total of 770 community members will be included in the baseline and follow-up questionnaire interviews. This means a random sample of at least 385 persons in Taraba State (northern Nigeria) and at least 385 persons in Cross River State (southern Nigeria), which will consist of 114 in the audio intervention group, 114 in the written material intervention group, and 157 in the control group in each region.

This RCT will have three arms: (1) an audio-delivered intervention group, (2) a written material intervention group, and (3) a control group. The sample size calculation is based on 2 calculations. The intervention group calculation is based on an estimate of the difference in knowledge improvement between the audio-delivered intervention and the written material intervention groups. We used data from a perception study in India. In this study, postintervention scores improved by 12.5% after a poster intervention and community meetings. We estimate that the effect of posters alone would be an increase of 10%. We want to be able to detect an improvement of at least 15% between the audio-delivered and the written material intervention groups. The sample size of the intervention group is therefore based on a proportion 1 of 10 (estimated percentage of improvement in knowledge of leprosy in the written intervention group) and a proportion 2 of 25 (ie, an improvement of 15% or more). With a power of 80%, a significance level of .05, and a 15% loss to follow-up, 114 participants are needed in each intervention group.

We expect an increase of 2% in the knowledge score in the control group. The control group calculation is therefore as follows: proportion 1:10, proportion 2:2, power 80%, significance .05, and 15% loss to follow-up, resulting in 157 participants.

A total of 25 people will be included in the participatory development of the material [41]. In each state, these will consist of 10 persons affected by leprosy and 2-3 community members. Semistructured interviews will be conducted until data saturation is reached.

### Description of the Intervention

The interventions consist of (1) an audio-delivered intervention and (2) a written or printed intervention (such as posters or flyers) for education on leprosy, awareness-raising, and stigma reduction.

We will compare the effect of the interventions with a control group. The audio-delivered and written content will be developed based on local beliefs, misconceptions, and fears about leprosy identified in the baseline study. This will be done using participatory approaches. A group of persons affected by leprosy and a few members of the community will be formed, who will be guided by a researcher to develop the messages and materials (participatory development). The key messages of the audio-delivered and written interventions will be the same. The materials will be developed in the main languages spoken in the study areas: Nigerian Pidgin (Cross River State) and Hausa (Taraba State). The majority (>80%) of our target group speaks either Nigerian Pidgin or Hausa, which will hence be the language used for this study. The audio-delivered intervention will be incorporated into Audiopedia [42]. Audiopedia’s website was designed to provide access to open knowledge foremost on health, livelihood, and well-being to both community-based organizations and nongovernmental organizations and individuals. Community-based organizations and nongovernmental organizations can benefit from using Audiopedia as part of their social and behavior change communication strategy, as it enables them to search, download, embed, and share audio files. Approximately 5000 audio clips, with a total runtime of 150 hours, are available in 11 languages. Audiopedia was optimized for search engines, thus making contents easy to find. Audiopedia also provides several technological solutions to make audio-based contents accessible to both literate and illiterate audiences, such as solar-powered audio players, mobile web applications for smart (feature) phones, and Wi-Fi hotspots that can stream audio-based contents without the need for internet connectivity, etc.

### Participant Recruitment and Follow-Up

Persons affected by leprosy and community members will be selected based on purposive sampling. They will develop the content of the interventions. This is therefore, ideally, a diverse group of people who represent multiple perspectives. We will select participants based on purposive sampling to ensure adequate representation of age, gender, and villages.

The intervention will be implemented in North and South Nigeria, 2 areas that are very different. Therefore, we will cluster-randomize the interventions to ensure comparable groups are included in both areas. There are three different “groups” in each site (North and South): (1) the audio-delivered intervention, (2) the written materials intervention, and (3) no intervention (control group).

We will select 3 LGAs in North and South Nigeria (6 in total); each LGA will have either the audio-delivered or the written intervention or be a control group. The three LGAs per area will be selected based on their similarity in terms of literacy rate and endemicity of leprosy.

**Figure 1:**
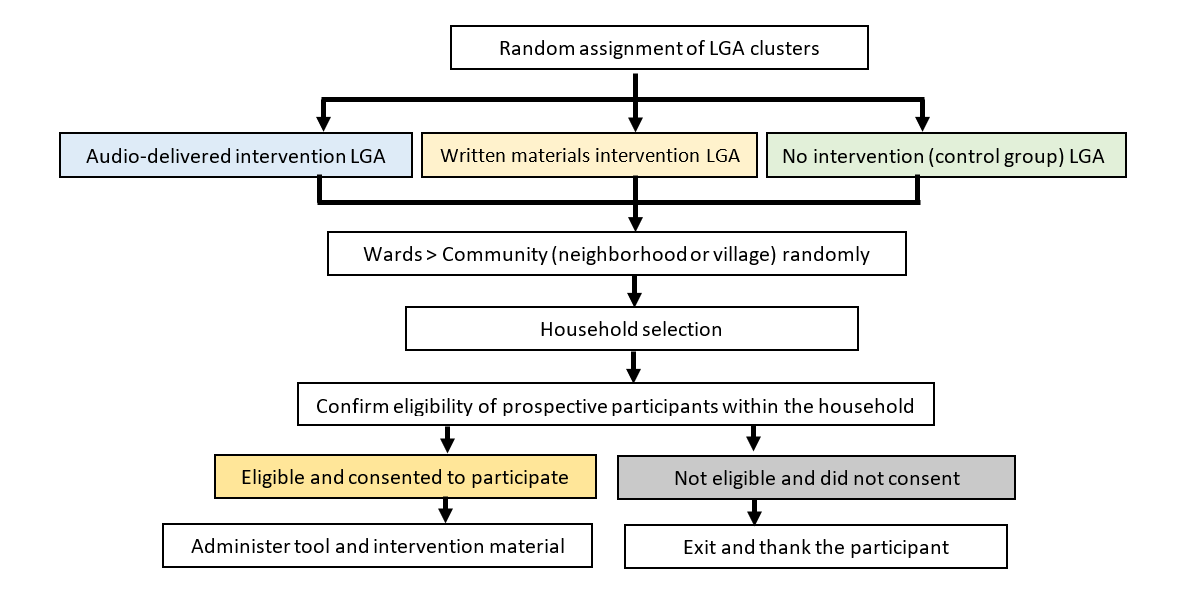
Study flow chart. LGA: local government area.

The interventions will be randomly allocated to clusters (LGAs) based on a random numbers list. This is a 2-stage random sampling. We will select a random sample of LGAs and a random sample of participants within each LGA. The participants in each LGA will be selected using the “spin the bottle” approach: a bottle will be spun in front of the most central place in the village (rural area) or neighborhood (urban area); the direction the bottle points at is the direction we start walking and counting. The first house to be included is selected by casting a die. Participant selection will be done by proportional sampling among the clusters. We will include the same participants at baseline and follow-up (a paired sample). This study protocol is reported in accordance with the SPIRIT statement (see checklist in Multimedia Appendix 1). The timeline schedule for participants during the study period is represented in Table 1, and a schematic description of the study flow chart is depicted in Figure 1.

**Table 1:** Study schedule for enrollment, intervention, and assessment.

Schedule				Study period and time point										
				Enrollment		Allocation		Postallocation						Close out
				t_–1_		t_0_ (month 0)		t_1_ (baseline)		t_2_ (intervention)		t_3_ (6 months follow-up)		t_4_
**Enrollment**														
	Eligibility screening		✓											
	Informed consent		✓											
	Randomization and allocation				✓									
**Intervention group**														
	Audio-delivered intervention								✓					
	Written								✓					
	Control (no intervention)													
**Assessments**														
	**Baseline variables**													
		KAP^a^, EMIC-CSS^b^, and SDS^c^					✓							
		RSES^d^ and L-ISMI^e^ scale					✓							
		CNA^f^					✓							
	**Outcome variables**													
		KAP, EMIC-CSS, and SDS									✓			
		RSES and L-ISMI scale									✓			
**Analysis**														
	Data analysis												✓	

^a^KAP: knowledge, attitudes, and practices.

^b^EMIC-CSS: Explanatory Model Interview Catalogue Community Stigma Scale.

^c^SDS: Social Distance Scale.

^d^RSES: Rosenberg Self-Esteem Scale.

^e^L-ISMI: Leprosy-adapted Internalized Stigma of Mental Illness.

^F^CNA: Communication Needs Assessment.

### Primary and Secondary End Points

The outputs of this study will be an audio-delivered intervention and written or printed health education material for education on leprosy, awareness-raising, and stigma reduction.

In addition, all 4 scales will be translated and cross-culturally validated in Nigerian Pidgin and Hausa languages as part of this study. The qualitative outcomes are knowledge and attitudes toward (persons affected by) leprosy (eg, local beliefs, fears, and misconceptions).

Outcome measures used to be assessed at baseline and follow-up are as follows:

- Demographic information: to disaggregate data by gender, the “knowledge, attitudes, and practices” (KAP) measure includes a form to collect demographic information.
- The KAP measure, as used in a study in India [16], covers 8 main topics: early symptoms, cause, mode of transmission, treatment, prevention, curability, contagiousness when on treatment, and prevention of disabilities. The KAP is a questionnaire and has not been formally validated as a scale (nor will it be used as a scale). We will translate and pilot test the KAP measure.
- Community stigma, using the EMIC-CSS (used in Nigeria but not validated in Nigerian Pidgin and Hausa yet [17]).
- Desired social distance toward persons affected by leprosy as a proxy for attitudes and fear, using the SDS (used among persons affected by leprosy in Nigeria but not validated in Nigerian Pidgin and Hausa yet [17]).
- Self-esteem and internalized stigma of persons affected by leprosy, using the RSES (used in Nigeria but not validated in Nigerian Pidgin and Hausa yet) and the leprosy-adapted ISMI (used among persons affected by leprosy and used in Nigeria but not formally validated yet [17]).

### Data Collection

#### Overview

We will use a mixed methods approach and collect qualitative data (in-depth interviews and focus group discussions [FGDs]) and quantitative data (the KAP measure, EMIC-CSS, SDS, communication needs assessment, RSES, and ISMI). We will also collect demographic information (including literacy levels) from each participant.

This study consists of the steps and phases outlined in the following subsections.

#### Step 1

None of the tools listed in step 2 have been validated in Nigerian Pidgin and Hausa yet; therefore, they will be cross-culturally validated before use. We will assess conceptual, item, semantic, operational, and measurement equivalence using a framework for cross-cultural equivalence testing based on the work of Herdman et al [43], Terwee et al [44], and Stevelink and van Brakel [45]. In addition, the interview and group discussion guides (outlined in step 2) will be pilot tested among a small sample of participants before use. The KAP measure will be translated and pilot-tested.

#### Step 2

A baseline study to assess the perceptions of community members using mixed methods will be conducted, consisting of both in-depth interviews and FGD sessions and questionnaires (KAP, EMIC-CSS, and SDS). A communication needs assessment will be conducted as part of the baseline study to determine the most appropriate mode of delivery of the audio intervention and the most appropriate duration and frequency of the audio intervention. We will also conduct a baseline study among the persons affected by leprosy group who will be involved in the development of the interventions. Self-esteem and internalized stigma will be assessed using in-depth interviews, RSES, and ISMI.

#### Step 3

The participatory development of the audio-delivered and written content of the interventions is based on the knowledge gaps, beliefs, misconceptions, fears, and community attitudes identified in the baseline study. We will use the “6 steps in quality intervention development (6SQuID) framework [46], consisting of (1) defining and understanding the problem and its causes, (2) identifying which causal or contextual factors are modifiable: which have the greatest scope for change and who would benefit most, (3) deciding on the mechanisms of change, (4) clarifying how these will be delivered, (5) testing and adapting the intervention, and (6) collecting sufficient evidence of effectiveness to proceed to a rigorous evaluation. The final step of 6SQuID (step 6) is part of step 5 of this study—evaluation. The group of persons affected by leprosy who play a leading role in the development of the messages and materials (participatory development) will codetermine both the content of the materials (based on the knowledge gaps, beliefs, misconceptions, fears, and community attitudes identified in the baseline study) and the mode of delivery (based on the most appropriate means of communication determined by the communication needs assessment conducted as part of the baseline study).

#### Step 4

Implementation of the interventions in the study areas will be done in 2 groups: one intervention group will receive the audio-delivered intervention, and the other intervention group will receive the written intervention (poster or flyer). A control group will not receive any intervention. The groups will be cluster-randomized.

#### Step 5

Step 5 involves evaluating the impact of (1) the intervention on the community and (2) developing the intervention on persons affected, using the same mixed methods as the baseline studies (described in step 2). It should be noted that the in-depth interviews at baseline are mainly conducted to get insight into specific fears, local beliefs, and misconceptions about leprosy among community members, as well as insight into the self-esteem and internalized stigma of persons affected by leprosy. At follow-up, the communication needs questionnaire will not be administered anymore. In addition, FGDs will be held with community members at baseline and follow-up in the 6 LGAs and with the persons affected by leprosy group. In addition to further exploring themes that arose during the in-depth interviews, additional questions will be asked (at follow-up) to get insight into, among other things, awareness of, experiences with, and exposure or access to the audio and written interventions; thoughts about content; mode of delivery and frequency; and strengths and points of improvement.

### Data Management and Data Analysis

Quantitative data collection will be done using electronic forms developed in the Open Data Kit, and all data will be securely stored in the cloud with access only to the research team. The recordings of the in-depth interviews and FGDs will be transcribed to the local languages, translated to English, and analyzed by 2 independent researchers using open, inductive coding and content analysis. Similar phrases with recurring themes will be coded in NVivo (QSR International). Quantitative data will be collected in the KoboCollect (Kobo Inc) mobile phone app. Data analysis will be done in the software package SPSS Statistics (SPSS Inc). Simple descriptive methods will be used to generate a demographic profile of the study sample. Differences between participants in the groups (audio, written materials, and control groups) will be evaluated using the Mann-Whitney *U* test or 1-tailed *t* test for continuous variables and the chi-square statistic for categorical variables. The mean (SD) or median (IQR), depending on the distribution of the data, of the total scores of the scales used will be calculated per intervention area. Stepwise multivariate regression with backward elimination will be done to examine what factors will have an independent effect on the outcomes. We will calculate the percentage change and corresponding 95% CI before and after the interventions are implemented and the statistical significance of this difference using a z test for differences between proportions. Effect sizes will also be calculated. If necessary, we will correct for differences in demographic information between study arms using quantile regression. We will compare the differences in follow-up assessment between the audio-delivered and written interventions. The knowledge gaps, beliefs, misconceptions, fears, etc identified in the baseline study will be summarized into a narrative review and will be used by the persons affected by leprosy group as input for the development of the content of the interventions. The most appropriate modes of delivery will be determined based on the communication needs assessment that will be conducted as part of the baseline study. The interventions will be developed using participatory methods.

Confidentiality and anonymity of data will be ensured in data collection, storage, analysis, and publication. Research assistants who will collect the data will be trained in data management, the maintenance of confidentiality, and ensuring privacy during data collection. Data will only be analyzed and shared with the Dutch and German researchers (outside of Nigeria) when they have been fully anonymized. The lead applicant will take full responsibility for ensuring the appropriate storage and security of data. Data will be kept for 5 years and destroyed after this time frame when no longer required.

### Ethical Considerations

Ethical approval has been obtained from the Health Research and Ethics Committee of the University of Nigeria Teaching Hospital, Ituku-Ozalla, Enugu (NHREC/05/01/2008B-FWA00002458-1RB00002323). In addition, appropriate clearance was sought and obtained from the respective ethics committees of both the Taraba and Cross Rivers State Ministries of Health. The confidentiality of the study participants and the data collected from them will be ensured.

Written informed consent (Multimedia Appendix 2) will be obtained from the study participants. The questionnaire will be translated into the local languages included in this study and back-translated to ensure the soundness of the translation process. The consent form explicitly states the right of the participant to refuse giving consent or withdraw from the study at any point. Also, he or she can decline to answer any question. Informed consent will be obtained from each participant before participation. Each participant will be given a copy of the participant information sheet and consent form to keep.

This RCT study was prospectively registered with and listed on the Pan African Clinical Trials Registry (PACTR202205543939385).

## Results

This study was funded in June 2022, and community member participant recruitment started in January 2023. Baseline data collection was completed by May 2023, with a total of 811 respondents. Participatory cocreation of the audio and written health education content began in July 2023, and the materials are currently under development. Intervention will be administered after pilot-testing, and the follow-up period will last for 6 months. Neither the implementation of the study intervention nor the data analysis have commenced as of the time of submission. Study results are expected in September 2024.

## Discussion

### Principal Considerations

In this study, we will apply mixed methods to compare the impact of audio-delivered versus written health education intervention materials and a control group without any intervention on the perception (knowledge, beliefs, and attitudes) of community members regarding leprosy. Through a participatory cocreation process, we will develop the content of the intervention materials alongside persons affected by leprosy and implement a 3-arm cluster RCT to evaluate the effect of the intervention. The effect of the cocreation process on internalized stigma among the affected persons will also be evaluated.

Developing context-specific behavioral change intervention activities is critical to addressing stigma in many leprosy-endemic communities where leprosy is highly stigmatized. The outcome of this intervention is expected to influence knowledge and, hopefully, improve the attitudes of community members toward leprosy, as well as improve self-esteem among persons affected by leprosy. In the long run, early leprosy case finding will be boosted following stigma reduction. To the best of our knowledge, this is the first study of this kind in Nigeria. The generated educational materials (both the audio and written content) are reusable products that may be adopted or used by the national program. The audio-delivered educational content will be freely available on the Audiopedia platform, licensed under Creative Commons (CC BY).

### Limitations

There are inherent limitations associated with cluster-randomized studies, such as cluster size variability. With the challenge of achieving uniform cluster sizes, variability in cluster size could impact the generalizability of the results. Additionally, there is a risk of contamination between clusters; however, this is minimal given the geographic distance between the study cluster locations.

The study sites were purposefully selected due to their similarity in having a high prevalence of leprosy and G2Ds; thus, the findings from this study may not be generalizable to the entire Nigerian populace.

### Conclusion

Outputs from the research will offer policy makers, national and regional program managers, and partners reliable evidence for a new approach toward stigma reduction activities, thereby contributing toward global triple zero leprosy efforts. Content generated provides pragmatic and contextual evidence-based tools for effective health education campaigns and awareness creation, especially among populations with low literacy levels, both in Nigeria and other low- and middle-income countries.

## Appendix

Multimedia Appendix 1 SPIRIT 2013 Checklist: Recommended items to address in a clinical trial protocol and related documents.

Multimedia Appendix 2 Participant information sheet and informed consent form.

Multimedia Appendix 3 Peer review report by Leprosy Research Initiative (LRI), Amsterdam, The Netherlands.

## Abbreviations

6SQuID: 6 steps in quality intervention development
EMIC-CSS: Explanatory Model Interview Catalogue Community Stigma Scale
FGD: focus group discussion
G2D: grade 2 disability
ISMI: Internalized Stigma of Mental Illness scale
KAP: knowledge, attitudes, and practices
LGA: local government area
RCT: randomized controlled trial
RSES: Rosenberg Self-Esteem Scale
SDS: Social Distance Scale
WHO: World Health Organization

## Data Availability

This is a study protocol manuscript. Deidentified research data that support the findings from this study will be made publicly available when the study is completed and published.
