# Supplementary material for: Participatory Development and Assessment of Audio-Delivered Interventions and Written Material and Their Impact on the Perception, Knowledge, and Attitudes Toward Leprosy in Nigeria: Protocol for a Cluster Randomized Controlled Trial
Source: JMIR Res Protoc. 2024 Jan 24;13:e53130. doi: 10.2196/53130 (PMC10851127; doi:10.2196/53130)
Supplement: Multimedia Appendix 1 [file resprot_v13i1e53130_app1.docx]

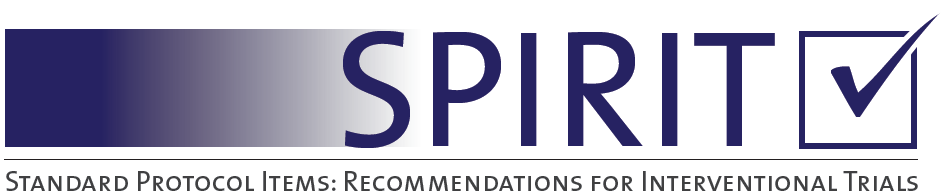


SPIRIT 2013 Checklist: Recommended items to address in a clinical trial protocol and related documents*

| Section/item | Item No | Description |
| --- | --- | --- |
| **Administrative information** | | |
| Title | 1 | Study Protocol for Participatory development of audio-delivered interventions and written material and assessment of their impact on the perception and knowledge of and attitudes towards leprosy: A cluster randomised trial in Nigeria |
| Trial registration | 2a | Pan African Clinical Trial Registry: PACTR202205543939385. |
|  | 2b | \| **Data category** \| **Information** \| \| --- \| --- \| \| Primary registry and trial identifying number \| Pan African Clinical Trials Registry  PACTR202205543939385 \| \| Date of registration in primary registry \| 18-May-22 \| \| Secondary identifying numbers \| Nil \| \| Source(s) of monetary or material support \| Leprosy Research Initiative (LRI) \| \| Primary sponsor \| Leprosy Research Initiative (LRI) \| \| Secondary sponsor(s) \| Nil \| \| Contact for public queries \| Joseph Chukwu, MBBS [+2348036678024] [joseph.chukwu@redaid-nigeria.org] \| \| Contact for scientific queries \| Ngozi Murphy-Okpala, FMCPH [+2348033705461] [ngozi.murphyokpala@redaid-nigeria.org]  56 Nza Street Independence Layout, Enugu, Nigeria \| \| Public title \| Audio and written interventions to improve the perception of leprosy \| \| Scientific title \| Participatory development of audio-delivered interventions and written material and assessment of their impact on the perception and knowledge of and attitudes towards leprosy: A cluster randomised trial in Nigeria \| \| Countries of recruitment \| Nigeria \| \| Health condition(s) or problem(s) studied \| Infections and Infestations, Skin and Connective Tissue Diseases \| \| Intervention(s) \| Active comparator: Audio Intervention  Active comparator: Written Intervention  Placebo comparator: No Intervention (Control group) \| \| Key inclusion and exclusion criteria \| Ages eligible for study: ≥18 years  Sexes eligible for study: Both  Accepts healthy volunteers: Yes  Inclusion criteria: Adults (≥ 18 years)  Exclusion criteria: Participants who do not speak Nigerian Pidgin or Hausa will be excluded. Persons unable or unwilling to give informed consent will be excluded. \| \| Study type \| Interventional  Allocation: randomized  Intervention model: parallel assignment  Masking: Open-label (Masking Not Used)  Primary purpose: Education /Training \| \| Date of first enrolment \| Jan-23 \| \| Target sample size \| 770 \| \| Recruitment status \| Recruiting \| \| Primary outcome(s) \| Knowledge, attitudes and practices using the KAP measure; Community stigma, using the EMIC Community Stigma Scale (EMIC-CSS); Social distance towards persons affected by leprosy using the Social Distance Scale (SDS) \| \| Key secondary outcomes \| Self-esteem and internalised stigma of persons affected by leprosy, using Rosenberg’s self-esteem scale \| |
| Protocol version | 3 | 31st May, 2023. Version 1. |
| Funding | 4 | Funded by a research grant from the Leprosy Research Initiative Foundation [FP_22\15]. |
| Roles and responsibilities | 5a | - Ngozi Murphy-Okpala, Medical department, RedAid Nigeria, Enugu, Nigeria - Tahir Dahiru, Medical department, Leprosy and TB Relief Initiative Nigeria, Jos, Nigeria - Anna van ’t Noordende, Technical Department, NLR, Amsterdam, The Netherlands - Carolin Gunesch, Department of Medical and Social Projects, DAHW-German Leprosy and TB Relief Association, Wurzburg, Germany - Joseph Chukwu, Medical department, RedAid Nigeria, Enugu, Nigeria - Charles Nwafor, Medical department, RedAid Nigeria, Enugu, Nigeria - Suleiman Abdullahi, Medical department, Leprosy and TB Relief Initiative Nigeria, Jos, Nigeria - Chukwuma Anyaike, National Coordinator, National Tuberculosis and Leprosy Control Programme, Federal Ministry of Health, Abuja, Nigeria - Ugochinyere Angelic Okereke, PhD student, Department of Art Education, University of Nigeria, Nsukka - Anthony Meka, Medical department, RedAid Nigeria, Enugu, Nigeria - Chinwe Eze, Medical department, RedAid Nigeria, Enugu, Nigeria - Okechukwu Ezeakile, Medical department, RedAid Nigeria, Enugu, Nigeria - Ngozi Ekeke, Medical department, RedAid Nigeria, Enugu, Nigeria. |
|  | 5b | Leprosy Research Initiative Foundation  1090 HA Amsterdam  The Netherlands |
|  | 5c | The funder does not have any direct influence over the study design and will not be part of the implementation.  There was an independent Steering committee (SRC) responsible for assessing the proposed study methodology including the appropriateness of proposed study design, methodology of data collection, data management, data analysis, and interpretation of data. The writing of the study report and decision to submit the report for publication is solely at the discretion of the researchers. |
|  | 5d | Composition, roles, and responsibilities of the coordinating centre, steering committee, endpoint adjudication committee, data management team, and other individuals or groups overseeing the trial, if applicable (see Item 21a for data monitoring committee)  The Steering committee (SRC) was responsible for assessing the appropriateness of proposed study design, methodology of data collection, data management, data analysis, and interpretation of data.  The coordinating centre where the principal investigator is domiciled will be responsible for the entire project management, which entails overseeing the overall project implementation and budget. Will also be responsible for coordinating the other co-applicants and relevant government staff for successful execution of planned activities. |
| **Introduction** | | |
| Background and rationale | 6a | Leprosy is an infectious disease caused by M. Leprae and has been stigmatized since ancient times. Stigma can occur at different levels, for example at intrapersonal, interpersonal, community, institutional and structural level. Many persons affected by leprosy experience negative consequences of their condition. Perception, which refers to how individuals or groups “see” an object, person, event or institution, is an important driver of stigma. The origin of stigma lies in public perceptions about people who are stigmatized. Perception comprises knowledge, beliefs and attitudes which are in turn influenced by personal factors (e.g., personality, experience) and environmental factors (e.g., culture, religion). Leprosy-related stigma is mainly caused by fear of contagion, (fear of) external manifestations and disabilities, religious and cultural beliefs, misconceptions, and a lack of knowledge. Access to appropriate health-information is an essential step in the fight against stigma and discrimination. However, written materials are not suitable for populations with low educational levels. To make health-information accessible to people who are illiterate in many leprosy endemic communities of the global south (especially women and girls), it is important to provide information through modalities other than printed materials. It is believed that audio-delivered interventions would be more suitable for illiterate and low-literate persons. Audio-delivered interventions, for example radio, have been shown to be successful for different stigmatized conditions like HIV, leprosy and albinism.  This study aims to assess the impact of an audio-delivered intervention on the perception (knowledge, beliefs, attitudes) of community members regarding leprosy by comparing an audio-delivered intervention with written health education. The study will be conducted in Nigeria. |
|  | 6b | The study will use a mixed-methods cross-sectional study design for the intervention development, and a three-arm cluster randomised trial for the intervention implementation and evaluation, comprising:   1. Baseline assessments of knowledge, attitudes, perceptions and fears of community members in order to develop the audio-delivered content and written material. 2. Baseline assessment of self-esteem and internalized stigma of persons affected by leprosy. 3. Participatory development of the audio-delivered content and written material by persons affected by leprosy. 4. Pilot and implementation of the interventions in Nigeria.   This will be done among different groups who will be compared (control group, audio-intervention group, written material group). Groups will be selected by cluster randomization. |
| Objectives | 7 | The primary objective is:  To assess the impact of audio-delivered and written health education on perception of leprosy  While the secondary Objectives are:  i. To investigate the perception (local beliefs, knowledge, attitudes) of community members towards leprosy and persons affected by leprosy  ii. To investigate whether there is a difference in impact on perception between participants who have received audio-delivered health education and those who have received written health education, with specific reference to gender differences and differences between rural and urban areas.  iii. To assess the impact of the participatory development of the audio and written interventions on empowerment and internalized stigma of persons affected by leprosy who developed the interventions. |
| Trial design | 8 | We will conduct a cross-sectional study (intervention development) and a three-arm cluster randomised trial (intervention implementation and evaluation). The three arms consist of:  (1) an intervention group who will receive the audio-delivered intervention,  (2) an intervention group who will receive the written intervention (poster/flyer), and  (3) a control group who will not receive any intervention. |
| **Methods: Participants, interventions, and outcomes** | | |
| Study setting | 9 | The community-based study will be carried out in six Local Government Areas (LGAs), three each in Cross River State (Boki, Calabar-South, and Obubra) and Taraba State (Jalingo, Yorro and Zing). The three LGAs per area will be selected based on similarity in terms of literacy rate and prevalence of leprosy.  Cross River is located in southern Nigeria while Taraba is in the north. The total population of the study area is 940,540 estimated from the 2006 census with 542,494 in Cross River and 398,046 in Taraba states. Both states have been selected because of the high prevalence of leprosy and grade-2-disability (G2D). |
| Eligibility criteria | 10 | Inclusion Criteria:  Only individuals of 18 years or older will be included.  Exclusion criteria:  Participants who do not speak Nigerian Pidgin or Hausa will be excluded. Persons unable or unwilling to give informed consent will be excluded. |
| Interventions | 11 | The interventions consist of:  (1) an audio-delivered intervention,  (2) a written/printed intervention (such as posters/flyers)  for education on leprosy, awareness raising and stigma reduction. We will compare the effect of the interventions with a control group.  The audio-delivered and written content will be developed based on local beliefs, misconceptions and fears about leprosy identified in the baseline study. This will be done using participatory approaches. A group of persons affected by leprosy and a few members of the community will be formed, who will be guided by a researcher to develop the messages and materials (participatory development). The key messages of the audio-delivered and written interventions will be the same. The materials will be developed in the main languages spoken in the study areas: Nigerian Pidgin (Cross-River) and Hausa language (Taraba). The majority, >80% of our target group, speaks either Nigerian Pidgin or Hausa language, and hence, will be the language used for this study.  The audio-delivered intervention will be incorporated in Audiopedia website, www.audiopedia.io.  The participatory co-development of the audio and written materials with persons affected by leprosy is also an intervention, and we will access assess the impact of the participatory development on the internalized stigma of these affected persons who co-developed the interventions. |
|  | 11b | In keeping with voluntary participation in research, participants are free to discontinue being part of the study at any time. |
|  | 11c | Adequate information will be provided to all potential participants to encourage willing participation and adherence. |
|  | 11d | Not applicable. |
| Outcomes | 12 | Key outcome measures to be assessed (at baseline and follow-up) are, difference in:   - Knowledge, attitudes and practices using the KAP measure (the KAP questionnaire covers eight main topics: early symptoms, cause, mode of transmission, treatment, prevention, curability, contagiousness when on treatment and prevention of disabilities). - Community stigma, using the EMIC Community Stigma Scale (EMIC-CSS) - Desired social distance towards persons affected by leprosy as a proxy for attitudes and fear, using the Social Distance Scale (SDS) - Self-esteem of persons affected by leprosy using Rosenberg’s self-esteem scale - Internalised stigma of persons affected by leprosy using the leprosy-adapted Internalized Stigma of Mental Illness scale (ISMI) |
| Participant timeline | 13 | The entire study duration is 2 years (24 months). Active participant recruitment will occur within 6-9months. Intervention development and piloting will last for the next 3 months after which the intervention will be administered. Then post-intervention assessment will be conducted 6 months after administering the intervention. |
| Sample size | 14 | The sample size for the various components of the study are as follows:   1. 200 community members (100 for each language/in each study area) will be included in the cross-cultural validation of the EMIC-CSS, SDS; while 100 persons affected by leprosy (50 for each language/in each study area) will be included for the cross-cultural validation of ISMI and Rosenberg’s self-esteem scale (see below). 2. A total of 760 community members will be included in the baseline and follow-up questionnaire interviews. This means a random sample of at least 385 persons in Taraba state (northern Nigeria) and at least 385 persons in Cross-River state (southern Nigeria); which will consisting of: 114 in the audio intervention group, 114 in the written material intervention group and 157 in the control group) in each region.   The sample size calculation is based on two calculations. The intervention group calculation is based on an estimate of the difference in knowledge improvement between the audio-delivered intervention and the written material intervention groups. We used data from a perception study in India, in this study, post-intervention scores had improved by 12.5% after a poster intervention and community meetings. We estimate that the effect of posters alone would be an increase of 10%. We want to be able to detect an improvement of at least 15% between the audio-delivered and the written material intervention groups. The sample size of the intervention group is therefore based on a proportion 1 of 10 (estimated percentage of improvement in knowledge of leprosy in the written intervention group) and proportion 2 of 25 (i.e., an improvement of 15% or more). With a power of 80%, significance level 0.05 and 15% loss to follow-up, 114 participants are needed in each intervention group. |
| Recruitment | 15 | The intervention will be implemented in North and South Nigeria – two areas that are very different. Therefore, we will cluster-randomise the interventions to ensure comparable groups are included. Both selected study areas are leprosy-endemic communities. Adequate time will be allowed for continuous recruitment at the communities until the sample size is achieved. We will include the same participants at baseline and follow-up (paired sample). Persons affected by leprosy and community members will be selected based on purposive sampling to ensure adequate representation of age, sex and villages. |
| **Methods: Assignment of interventions (for controlled trials)** | | |
| Allocation: |  |  |
| Sequence generation | 16a | The sequence generation was to assign numbers to the 6 study LGAs (3 LGAs in each region of northern and southern Nigeria). An independent (visually impaired) person who is not part of the study used simple balloting method to assign each LGA to the respective audio intervention or written intervention or control cluster. |
| Allocation concealment mechanism | 16b | Not applicable |
| Implementation | 16c | This is a cluster-randomized study hence, intervention assignment is the cluster-level rather than individual. |
| Blinding (masking) | 17a | Not applicable |
|  | 17b | Not applicable |
| **Methods: Data collection, management, and analysis** | | |
| Data collection methods | 18a | We will use a mixed-methods approach and will collect qualitative data (in-depth interviews and focus group discussions) and quantitative data (the KAP measure, the EMIC-CSS, SDS, Communication Needs Assessment, Rosenberg’s self-esteem scale and the ISMI). We will also collect demographic information (including literacy levels) from each participant. Quantitative data will be collected in KoboCollect mobile phone app to promote data quality and completeness.  Although the original study tools have been used in Nigeria, none of them have been validated in Nigerian Pidgin and Hausa yet, therefore they will be cross-culturally validated before use. We will assess conceptual, item, semantic, operational and measurement equivalence using a framework for cross-cultural equivalence testing. |
|  | 18b | Participation in research is voluntary, but we will ensure all potential participants are sufficiently informed so as to willingly participate and remain compliant till study completion. |
| Data management | 19 | Quantitative data collection will be done using electronic forms developed in Open Data Kit (ODK) based KoboCollect mobile phone app, and all data will be securely stored in the cloud with access only to the research team. This ensures quality data collection by use of range checks and prevents missing or duplicate data.  Confidentiality and anonymity of data will be ensured in data collection, data storage, analysis and publication. Research assistants who will collect the data will be trained in data management, maintenance of confidentiality and ensuring privacy during data collection. |
| Statistical methods | 20a | Data analysis will be done in the software package SPSS Statistics. Simple descriptive methods will be used to generate a demographic profile of the study sample. Differences between participants in the groups (audio, written materials and control groups) will be evaluated using the Mann-Whitney U test or t-test for continuous variables and the chi-square statistic for categorical variables.  The mean with SD or median with interquartile range (depending on the distribution of the data) of the total scores of the scales used will be calculated per intervention area. Stepwise multivariate regression with backward elimination will be done to examine what factors will have an independent effect on the outcomes. We will calculate the % change and corresponding 95%CI before and after the interventions are implemented and the statistical significance of this difference using a Z-test for differences between proportions. Effect sizes will also be calculated. If necessary, we will correct for differences in demographic information between study arms using quantile regression. We will compare the differences in follow-up assessment between the audio-delivered and written interventions.  The recordings of the in-depth interviews and FGDs will be transcribed to the local languages, translated to English and analysed by independent researchers using open, inductive coding and content analysis. Similar phrases with recurring themes will be coded in the software programme, Nvivo. |
|  | 20b | The intervention effect will be analysed with specific reference to gender differences and differences between rural and urban areas. |
|  | 20c | Not applicable. |
| **Methods: Monitoring** | | |
| Data monitoring | 21a | As this is not a clinical trial, a data monitoring committee (DMC) is not needed. However, several measures have been put in place to ensure proper data management. This includes confidentiality and anonymity of data which will be ensured in data collection, data storage, analysis and publication. Research assistants who will collect the data will be trained in data management, maintenance of confidentiality and ensuring privacy during data collection. |
|  | 21b | Not applicable |
| Harm | 22 | This study only involves minimal risk from personal information provided. No physical harm is envisaged from participating in this study. |
| Auditing | 23 | Not applicable |
| **Ethics and dissemination** | | |
| Research ethics approval | 24 | Ethical approval was obtained from the Heath Research and Ethics Committee of the University of Nigeria Teaching Hospital (UNTH), Ituku-Ozalla, Enugu; *NHREC*/05/01/2008B-FWA00002458-1RB00002323.  In addition, appropriate clearance was sought from the respective Ethics Committee of both Taraba and Cross Rivers State Ministry of Health. |
| Protocol amendments | 25 | In the event of any modification to the study protocol, the Heath Research and Ethics Committee of the University of Nigeria Teaching Hospital (UNTH), Ituku-Ozalla, Enugu will be duly informed to seek approval. Any subsequent update will be communicated with the trial registry as well. |
| Consent or assent | 26a | Informed consent will be obtained from each participant prior to participation by the trained research assistants, after clearly communicating the aims, benefits and risks of participating in the study. The consent form explicitly states the right of the participant to refuse giving consent or withdraw from the study at any point. Also, he/she can decline to answer any question. Detailed contact information of the principal investigator is provided in the information sheet. Each participant will be given a copy of the participant information sheet and consent form to keep. |
|  | 26b | Not applicable |
| Confidentiality | 27 | Confidentiality and anonymity of data will be ensured in data collection, data storage, analysis and publication. Research assistants who will collect the data will be trained in data management, maintenance of confidentiality and ensuring privacy during data collection. |
| Declaration of interests | 28 | Authors declare no financial or competing interests. |
| Access to data | 29 | The lead applicant will take full responsibility for ensuring the appropriate storage and security of data. All data will be securely stored in the cloud with access only to the research team. Data will be kept for five years and will be destroyed after this time frame when no longer required. Data will only be analysed and shared with the Dutch and German researchers (outside of Nigeria) when they have been fully anonymized. |
| Ancillary and post-trial care | 30 | Not applicable |
| Dissemination policy | 31a | Study results will be published in peer-reviewed, open-access journals. Findings will also be presented at conferences. In addition, we intend to share our findings across the International Federation of Anti-Leprosy Associations (ILEP) network as well as with government officials in Nigeria through a dissemination meeting. |
|  | 31b | Authorship for this publication will be based on the International Committee of Medical Journal Editors (ICMJE) guidelines for defining the Role of Authors and Contributors.  There are no plans nor intended use of professional writers. |
|  | 31c | The full study protocol will be published in a peer-reviewed open-access journal. |
| **Appendices** | | |
| Informed consent materials | 32 | Consent form attached as appendix. |
| Biological specimens | 33 | Not applicable |
