# Supplementary material for: Participatory Development and Assessment of Audio-Delivered Interventions and Written Material and Their Impact on the Perception, Knowledge, and Attitudes Toward Leprosy in Nigeria: Protocol for a Cluster Randomized Controlled Trial
Source: JMIR Res Protoc. 2024 Jan 24;13:e53130. doi: 10.2196/53130 (PMC10851127; doi:10.2196/53130)
Supplement: Multimedia Appendix 2 [file resprot_v13i1e53130_app2.docx]

## APPENDIX

### Annex 1 – Participant Information Sheet and Informed Consent Form

**Principle Investigator:** Dr Ngozi Murphy-Okpala, RedAid Nigeria

**Co-Investigator:** Dr Tahir Dahiru, Leprosy and TB Relief Initiative Nigeria

**Sponsor:** Leprosy Research Initiative (LRI)

**Project Title:** Audio and written interventions to improve the perception of leprosy

This Informed Consent Form has two parts: 1. **Information Sheet** (to share information about the study with you). 2. **Certificate of Consent** (for signatures if you choose to participate). You will be given a copy of the full Informed Consent Form.

**Part I: Information Sheet**

**Introduction**

I am ………………………..(the RA to mention his/her name), working for ……..state TB and leprosy control programme and LTR or RedAid Nigeria. We are doing research on leprosy which is common in this LGA. I am going to give you information and invite you to participate in this study. You do not have to decide today whether or not you will participate in this study. Before you decide, you can talk to anyone you feel comfortable about the study. This consent form may contain words that you do not understand. Please ask me to stop as we go through the information, and I will take time to explain. If you have questions later, you can ask me or my colleague.

**Purpose of the research**

Leprosy is a disease that continues to affect people in this community, it is a disease that if not detected early and treated can result in disability which in turn can lead to stigma, discrimination, and social exclusion of the people affected and their families. We want to find ways to reduce and stop this from happening. We believe that you can help in achieving this by telling us what you know about the disease. We want to learn what people (in this community) know about causes of leprosy, and how the disease is transmitted. We want to learn about people’s beliefs and attitudes towards people affected by leprosy and their families. We also want to know if use of audio-delivered health messages can have impact on this perception of leprosy compared to written health education messages.

**Type of Research Intervention**

This research will involve your participation in interviews before or after the use of audio-delivered health messages or written health messages on leprosy or no message at all. The interview will take about one hour.

**Participant Selection**

You have been chosen to participate in this study because you are a member of this community and we seek to understand how members of this community think about leprosy and persons affected by leprosy.

**Voluntary Participation**

Your participation in this study is entirely voluntary. It is your choice whether to participate or not. If you choose not to participate no penalty will be meted on you. You may change your mind later and stop participating even if you agreed earlier.

**Procedures**

We are asking you to help us learn more about leprosy in your community and if you accept, you may be asked to participate in an in-depth interview, focus group discussion and/or respond to a questionnaire.

***For in-depth interviews and focus group discussions:***

*You are invited to participate in an interview with me (or take part in a focus group discussion with 7-8 other community members). I will guide this discussion. The questions will be about leprosy in your community, what causes it, how it is spread, beliefs, and how people affected by leprosy and their family members are treated in the community. I want to ask you to share personal beliefs, practices or stories about leprosy and people affected by leprosy and their family members.*

*If you do not wish to answer any of the questions during the interview or discussion, you may say so and the interviewer will move on to the next question. The interview and the discussion will be audio-recorded, but no-one will be identified by name. The audio file will be saved securely in a computer file accessible to the investigators alone. The information recorded is confidential, and no one else except the research team will have access to them. The recordings will be transcribed afterwards, and destroyed after 10 years from the interview date.*

***For questionnaire:***

*A questionnaire will be provided which will be read to you and I will write down your answer/response.*

*If you do not wish to answer any of the questions included in the survey, you may skip them and move on to the next question. The information recorded is confidential, your name will not be included on the forms, only a number will identify you, and no one else except the research team will have access to your survey.*

**Duration**

The research takes place over 24 months in total. The interview session will last for about one hour.

**Risks**

There is a risk that you may share some personal or confidential information by chance, or that you may feel uncomfortable talking about some of the topics. However, we do not wish for this to happen. You do not have to answer any question or take part in the discussion/interview/survey if you feel the question(s) are too personal or if talking about them makes you uncomfortable.

**Benefits**

There will be no direct benefit to you, but your participation is likely to help us find out more about how people’s perception on leprosy, develop and share messages on leprosy and stigma against persons affected by leprosy or their families which will contribute to the elimination of the disease and its consequences in Nigeria and the world.

**Confidentiality**

We will ensure confidentiality and anonymity of the information you will provide. We will not be sharing information about you to anyone outside of the research team. The information that we will collect from this research project will be kept private. Any information about you will have a number on it instead of your name. Only the researchers will know what your number is, and we will lock that information up with a lock and key.
**Sharing the Results**

Nothing that you tell us today will be attributed to you by name. The knowledge that we get from this research will be shared with you and your community before it is made widely available to the public. There will be small meetings in the state and following the meetings, we will publish the results so that other interested people may learn from the research.

**Right to Refuse or Withdraw**

You do not have to take part in this research if you do not wish to do so. You may stop participating in the at any time that you wish. I will give you an opportunity at the end of the interview/discussion to review your remarks, and you can ask to modify or remove portions of those, if you do not agree with my notes or if I did not understand you correctly.

**Who to contact**

If you have any questions, you can ask them now or later. If you wish to ask questions later, you may contact the principal investigator: Dr Ngozi Murphy-Okpala, [ngozi.murphyokpala@redaid-nigeria.org](mailto:ngozi.murphyokpala@redaid-nigeria.org).

This proposal has been reviewed and approved by the Research Ethical Review Committee of University of Nigeria, which is a committee whose task it is to make sure that research participants are protected from harm. The study has also been approved by the respective as well as the Ministry of Health in both Cross-River and Taraba states in each study location.

You can ask me any more questions about any part of the research study, if you wish to.

Do you have any questions?

**Part II: Certificate of Consent**

**Note:** A researcher or the person going over the informed consent must sign each consent. Participants who cannot write should use thumb print as signature.

I have been invited to participate in a research study about participatory development of audio-delivered interventions and written material and assessment of their impact on the perception and knowledge of and attitudes towards leprosy: a cluster randomized trial in Nigeria.

**(This section is mandatory)**

*I have read the foregoing information, or it has been read to me. I have had the opportunity to ask questions about it and any questions I had, have been answered to my satisfaction. I consent voluntarily to be a participant in this study*

**Print Name of Participant_____________________________________________________**

**Signature of Participant ___________________ Date ___________________________**

**Day/month/year**

***If illiterate^1^***

**I have witnessed the accurate reading of the consent form to the potential participant, and the individual has had the opportunity to ask questions. I confirm that the individual has given consent freely.**

**Print name of witness__________________*___________* Thumb print of participant**

**Signature of witness ____________________________ Date ________________________**

**Day/month/year**

**Statement by the researcher/person taking consent**

I have accurately read out the information sheet to the potential participant, and to the best of my ability made sure that the participant understood that one of the following will be done:

1. In-depth interview

2. Focus group discussion

3. Survey/questionnaire

4. Health education messages/ materials will be provided

***I confirm that the participant was given an opportunity to ask questions about the study, and all the questions asked by the participant have been answered correctly and to the best of my ability. I confirm that the individual has not been coerced into giving consent, and the consent has been given freely and voluntarily.***

**A copy of this ICF has been provided to the participant.**

**Print Name of Researcher****/person taking the consent______________________________**

**Signature of Researcher__________________________ Date __________________________**

A literate witness must sign (if possible, this person should be selected by the participant and should have no connection to the research team). Participants who are illiterate should include their thumb print as well.
