# Supplementary material for: Participatory Development and Assessment of Audio-Delivered Interventions and Written Material and Their Impact on the Perception, Knowledge, and Attitudes Toward Leprosy in Nigeria: Protocol for a Cluster Randomized Controlled Trial
Source: JMIR Res Protoc. 2024 Jan 24;13:e53130. doi: 10.2196/53130 (PMC10851127; doi:10.2196/53130)
Supplement: Multimedia Appendix 3 [file resprot_v13i1e53130_app3.pdf]

Full Proposal 2022
External Reviewers

Reference: FP_22\15
Project Title: Participatory development of audio-delivered interventions and written material and their impact on the perception and knowledge of and attitudes towards leprosy: a cluster randomised trial in Nigeria
Proposed Start Date: 01/02/2022
Applicant: Ngozi Murphy-Okpala
Organisation: RedAid Nigeria
Value Sought: €143,346.20

Reviewer 1:

Criteria	Reviewer Score<br>1=Poor, 2= Fair, 3=Good, 4=Very Good, 5=Excellent	Max Score
**Research question: Importance**	3	5
**Research question: Originality**	4	5
**Research question: Clarity**	4	5
**Feasibility: Research group**	3	5
**Feasibility: Facilities**	4	5
**Feasibility: Possibility of answers/results**	4	5
**Methodology: Design**	4	5
**Methodology: Sampling**	4	5
**Methodology: Data collection methods**	4	5
**Methodology: Analysis**	4	5
**Expected scientific impact**	3	5
**Likelihood of translating the results in daily practice**	4	5
**Total score**	**Your score: 45**	**Max score: 60**
**FTE for personnel national**	Too Much	The budget for national personnel takes about 32% of the entire budget. This is too high and should be reviewed downwards. I believe this research will be coducted as part of other projects being implemented by RedAid as such should be charged as a percentage of their time
**FTE for personnel international**	Adequate	

**Travel**	Adequate	
**Equipment**	Adequate	
**Running costs**	Adequate	
**Other costs**	Adequate	
**Total budget**	Adequate	
**What points are in favour of this proposal?**	While research comparing audio-deliverred interventions and written information may not be entirely new, I find its use in leprosy innovative. The research question is clear and the timeline seems quite feasible for completing the project.<br><br>The study adresses key themes - zero transmission and zero stigma and discrimination. The concept is well-thought-out and the proposal is well articulated. The project title is also suitable for the proposed study. The contents of the proposal indicate thorough review of the literature and a great deal of originality. The study method is well spelt-out and the choice of two study sites with some unique qualities is equally commendable.<br><br>Results could be scalable and have a positive impact on practice.	
**What are the critical reflections and points for improvement for this proposal?**<br>Max score:	The research should be clear with regards to the method of delivery of the audio message. This is not quite stated in the proposal if they would apply solar powred radios as promoted by audiopedia or they intend to apply mobile phones. Either choice would have implications on how the research will be implemented and how results could be scaled.<br><br>Rsearchers need to ensure literacy levels in all three arms of the research are similar as this is a key cofounding factor. They need to clealry state how this will be ensured<br><br>The protocol states it could deploy Nvivo, Atlas.ti or MAXDA. I think the researchers need to be specific on which particular qualitative software will be deployed for the analysis of qualitative data.<br>Consider involving community members in the development of the audio and written contents.<br>Ethical clearance obatined from UNTH is not enough. Ethhical clearance needs to be obtained from the relevenat institutions in Taraba and Cross RIver State governments.	

LEPROSY RESEARCH INITIATIVE

Reviewer 2:

Criteria	Reviewer Score<br>1=Poor, 2= Fair, 3=Good, 4=Very Good, 5=Excellent	Max Score
**Research question: Importance**	4	5
**Research question: Originality**	4	
**Research question: Clarity**	3	
**Feasibility: Research group**	2	
**Feasibility: Facilities**	4	
**Feasibility: Possibility of answers/results**	2	
**Methodology: Design**	3	
**Methodology: Sampling**	4	
**Methodology: Data collection methods**	4	
**Methodology: Analysis**	3	
**Expected scientific impact**	3	5
**Likelihood of translating the results in daily practice**	3	5
**Total score**	**Your score: 39**	**Max score: 60**
**FTE for personnel national**	Too Much	
**FTE for personnel international**	Adequate	
**Travel**	Adequate	
**Equipment**	Adequate	
**Running costs**	Too Much	
**Other costs**	Adequate	
**Total budget**	Too Much	
**What points are in favour of this proposal?**	The importance of identifying the best modalities for improving the perception of leprosy, reducing stigma and discrimination is great. The potential scientific values of this study to contribute to that issue is limited.<br><br>Topic is very important<br>Local focus is strong<br>Local leadership is strong<br>Team is very strong<br>Participatory commitment	
**What are the critical reflections and points for improvement for this proposal?**	This proposal has gone to great effort to demonstrate scientific quality through traditional quantitative mantras (cluster randomised controlled with great attention to sample size and analysis), but has not provided fundamental explanation of what the interventions will be. Operationalisation is too limited. Audio delivered interventions are not	

	operationalised in any way. The reason for this appears to be a focus on participatory and consumer directed content, which is great to hear and appropriately suggested, but which would therefore suggest that qualitative methods would be far more appropriate. Now it is undermined by the rigid quantitative methods and tools. If you are genuinely going to listen to people affected by leprosy and have them decide on mode (TV? Radio? Smartphone? AudiopediaDdevice? Loudspeaker? Town Crier?), duration (30 seconds? 3 hours?), content (Informational? Factual? Attitudinal? Medical?), frequency (daily? monthly?), etc., you can not pre-empt what your analysis and design and tools will be.<br><br>The audiopedia tool seems poorly targeted (and based on the website and facebook page) seems likewise lacking in information about suitability of content and efficacy.<br><br>It would seem that a major limitation to impact is that "Audiopedia" (which the researchers mention repeatedly) is expressly focussed on women. What will be the impact on 50% of your potential population if the tool you use for dissemination is only for women and focused on women? What sort of bias will that result in?
